# Achieving Continuous Self‐Powered Energy Conversion‐Storage‐Supply Integrated System Based on Carbon Felt

**DOI:** 10.1002/advs.202207033

**Published:** 2023-03-06

**Authors:** Ji Peiyuan, Li Qianying, Zhang Xuemei, Hu Yawen, Han Xiangyu, Zhang Dazhi, Hu Chenguo, Xi Yi

**Affiliations:** ^1^ Chongqing Key Laboratory of Soft Condensed Matter Physics and Smart Materials Department of Applied Physics Analytical and Testing Center Chongqing University Chongqing 400044 P. R. China; ^2^ School of Materials Science and Engineering Chongqing Jiaotong University Chongqing 400074 China; ^3^ Department of New Energy Power Evaluation and Research China Automotive Engineering Research Institute Co., Ltd Chongqing 401122 China

**Keywords:** energy harvesting, fast charging device, self‐powered system, supercapacitors, triboelectric nanogenerators

## Abstract

Efficient harvesting and storage of dispersed irregular energy from the environment are crucial to the demand for the distributed devices of the Internet of Things (IoTs). Here, a carbon felt (CF)‐based energy conversion‐storage‐supply integrated system (CECIS) that contains a CF‐based solid‐state supercapacitor (CSSC) and a CF‐based triboelectric nanogenerator (C‐TENG) is presented, which is capable of simultaneously energy storage and conversion. The simple treated CF not only delivers a maximal specific capacitance of 402.4 F g^−1^ but also prominent supercapacitor characteristics with fast charge and slow discharge, enabling 38 LEDs successfully lightened for more than 900 s after a wireless charging time of only 2 s. With the original CF as the sensing layer, buffer layer, and current collector of C‐TENG, the maximal power of 91.5 mW is attained. The CECIS shows a competitive output performance. The time ratio of the duration of supply energy to the harvesting and storage reaches 9.6:1, meaning that it is competent for the continuous energy application when the effective working time of C‐TENG is longer than one‐tenth of the whole day. This study not only highlights the great potential of CECIS in sustainable energy harvesting and storage but also lays the foundation for the ultimate realization of IoTs.

## Graphical abstract

Based on the outstanding capacity performance of carbon felt as an active electrode material for supercapacitors and the superior output performance of carbon‐felt‐based triboelectric nanogenerator (TENG), a carbon‐felt‐based self‐power system that contains a TENG and a supercapacitor for energy conversion and storage respectively is assembled to achieve continuous self‐power supply.

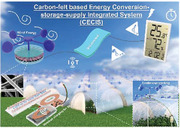

## 1 Introduction

Energy is a critical issue in the development of human civilization. Over the past three centuries, the overuse of coal, oil, and natural gas has caused the environmental quality seriously worsen. Not only that, the conversion, storage, and supply of conventional energy systems with orderly, point‐to‐point energy transmission characteristics is difficult to meet the needs of the Internet of Things (IoTs).^[^ ^1^ ^]^ Emerging self‐power devices and systems provide an opportunity to overcome this matter.^[^ ^2^ ^]^ These devices and systems adopt harvesting high‐entropy energy from the environment to address the energy supply needs of distributed, disordered, wireless small devices, thus improving the energy efficiencies in energy supply systems.^[^ ^3^, ^4^ ^]^

So far, a variety of new generations of devices and systems have been developed to harvest and convert high‐entropy energy including solar cells, wave energy, wind energy, and so on.^[^ ^5^, ^6^, ^7^, ^8^, ^9^, ^10^ ^]^ Among these, the triboelectric nanogenerator (TENG), which simultaneously harvests and converts irregular, scattered, and wasted mechanical energy to electrical energy, has received extensive attention. Existing works of TENG are mainly focused on driving some commonly used electronic devices by collecting and utilizing external energy to achieve power supply,^[^ ^11^, ^12^ ^]^ which inevitably relies on the environment. The intermittent environmental energy may cause the interruption of the power supply to the device. New types of energy conversion, storage, and supply systems with improved efficiency and reliability are therefore highly desirable. Some energy storage devices like capacitors have been added to meet the above‐desired performance, while the key building block for integrated systems is the matching between the TENG and energy storage unit.^[^ ^13^, ^14^, ^15^ ^]^ A paper‐based self‐charging device by integrating TENG and a supercapacitor on paper can be used as a self‐charging portable power source to drive an electronic thermometer and an electronic watch successfully.^[^ ^16^ ^]^ A fabric textile device integrates energy harvesting, storage, and sensing by integrating fiber‐like supercapacitors with fiber‐type TENG.^[^ ^17^ ^]^ Qiu et al. constructed a self‐powered device with an MXene‐based supercapacitor and a single‐electrode TENG, which can be useful for powering electronics without additional power sources.^[^ ^18^ ^]^

Although many works have combined TENG with supercapacitors to achieve continuous self‐power supply, limited by the unsatisfactory capacity of supercapacitors in the energy storage part, the continuous power supply time of common small electronic devices still needs to be improved. In addition, it requires a long operating time for TENG to charge the supercapacitors to achieve an effective external output voltage. Therefore, it still needs further exploration for continuous self‐powered systems on how to achieve long‐term driving of electronic devices while shortening the time of collecting external mechanical energy from the environment.

Carbon felt (CF) is a material composed of carbon fibers with high electrical conductivity, flexibility, stability, and low cost, which has been widely used in many fields.^[^ ^19^, ^20^ ^]^ In this work, the CF‐based energy conversion‐storage‐supply integrated system (CECIS) has been successfully assembled for continuous and highly reliable power applications. The CECIS is composed of a CF‐based solid‐state supercapacitor (CSSC) and a CF‐based TENG (C‐TENG) as the energy storage and conversion unit, respectively. The simply treated CF as the active electrode material for energy storage unit not only exhibits a maximal specific capacitance of 402.4 F g^−1^, but also reveals superior capacitive performance for symmetric, asymmetric, and solid‐state supercapacitors. Not only that, the fabricated CSSC enables 38 LEDs to successfully light for >900 s after a wireless charging time of only 2 s. The flexible CF can also simultaneously serve as the sensing layer, buffer layer, and current collector to simplify the structure of C‐TENG with a maximal power of 91.5 mW. Benefiting from the ideal capacitive behavior of CSSC and the outstanding output performance of C‐TENG, the as‐assembled CECIS exhibits excellent continuous output characteristics. A hygrothermograph has been lightened successfully for >18 min by CSSC after being charged by C‐TENG for less than 2 min. The time ratio of the duration of supply energy for driving the hygrothermograph to the harvesting and storage of energy reaches 9.6:1. That is, the CECIS can be faultlessly competent as a continuous and highly reliable power device just the effective working time of C‐TENG is longer than one‐tenth of the whole day. This work highlights the great potential of CECIS in sustainable energy harvesting and conversion and lays the foundation for the ultimate realization of IoTs.

## 2 Results and Discussion

### 2.1 Application Schematic and Main Output Features of CECIS

**Figure 1:**
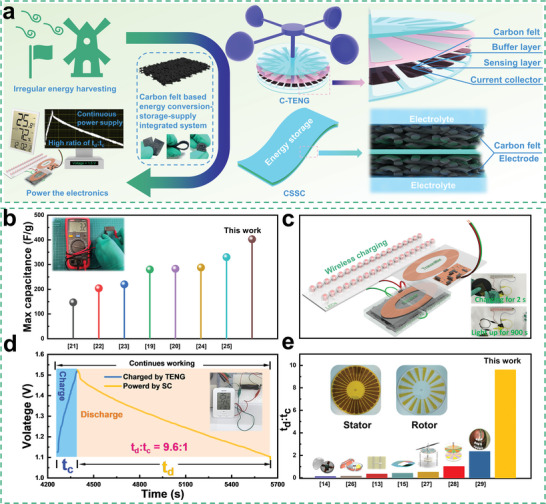
Schematic and main features of CECIS. a) Conceptual schematic of CECIS. b) The maximal specific capacitance of treated CF (named CFHK) compared with other works, the inset shows the corresponding resistivity tested with a multimeter. c) Schematic structure of the wireless charging system when using CFHK as the electrode for supercapacitors with fast charging and slow discharging behavior. The insets show the wireless charging process and light up the LEDs. d) Voltage‐time curve of CECIS at driving a hygrothermograph. The inset exhibits a running hygrothermograph. e) The comparison of *t*_d_:*t*_c_ with other similar works. The insets demonstrate the photographs of the stator and rotor.

The CECIS consists of two main components, a C‐TENG for harvesting and converting the irregular energy, a CSSC for energy storage and supply to power the electronics. CF with distinguished flexibility and conductivity acts as the key material in CECIS. As shown in **Figure** **1**a, CF can be simultaneously served as a buffer layer, sensing layer, and current collector of C‐TENG for better contact between the friction layers and to simplify the structure. Moreover, Figure 1b and inset reveal that the as‐treated CF (named CFHK) can be a superb supercapacitors electrode material with superior capacity. To be specific, the CFHK delivers a maximal specific capacitance of 402.4 F g^−1^ with clear advantages compared with the other works.^[^ ^19^, ^20^, ^21^, ^22^, ^23^, ^24^, ^25^ ^]^ Using the CFHK as the active electrode material, the CSSC reveals advanced capacitance performance with fast charging and slow discharging properties. 38 LEDs have been successfully lightened up for more than 900 s after a wireless charging time of 2 s (Figure 1c). Benefiting from the exceptional performance of C‐TENG and CSSC, the as‐assembled CECIS not only realizes remarkable energy conversion and storage ability but also achieves an efficient continuous power supply capability. The key parameter for evaluating the performance of the CECIS is the ratio of energy harvesting time (*t*_c_) to sustainable power supply time (*t*_d_). The typical voltage‐time curve of a CECIS‐driven hygrothermograph is revealed in Figure 1d. The value of *t*_c_:*t*_d_ is characterized and determined to be 9.6:1, indicating that only about one‐tenth of the day is required for energy harvesting to achieve continuous self‐powering throughout all day. Figure 1e depicts the value of *t*_c_:*t*_d_ for CECIS and other similar works. As a result, CECIS shows more than four times that of the others with clear advantages.^[^ ^13^, ^14^, ^15^, ^26^, ^27^, ^28^, ^29^ ^]^

### 2.2 Characterization and Application of Carbon Felt as Supercapacitor Material

**Figure 2:**
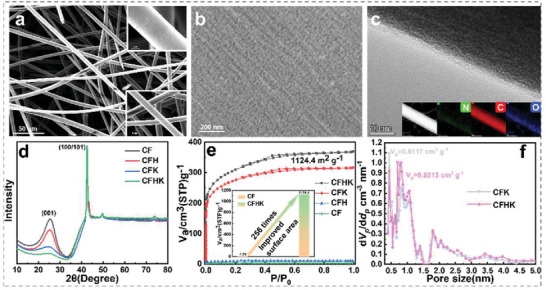
Characterization results of as‐prepared samples as the electrode material for supercapacitors. a,b) SEM images of CFHK under different magnifications. c) TEM images and elements mappings of CFHK(inset). d) XRD spectrum of CF, CFH, CFK, and CFHK. e) BET results of four samples, the inset reveals the specific surface area of CF and CFHK. f) the pore size and distribution of CFK and CFHK.

The morphology, lattice information, specific surface area, and pore size distribution have been characterized and the results are demonstrated in **Figure** **2**. Figure 2a exhibits the SEM images of the as‐prepared CFHK with an intersecting fiber structure. The overall morphology is complete without obvious cracks or breakages, thus providing a stable conductive path for the transportation of the electrons. Figure 2b reveals the uniformly distributed cracks on the surface of the fibers, which are conducive to the efficient penetration of the electrolyte. Besides, the hydrophilicity has also been improved. Compared with the original CF, CFHK can be better wetted when immersed in KOH solution, as illustrated in Movie S1, Supporting Information. It depicts the TEM images on the edge of the carbon fiber in Figure 2c. Besides, C, O, and N elements can be observed from element mappings results in the insets, which are corresponding to the EDS results, as displayed in Figure S2. The used CF in this work is made from polyacrylonitrile and the chemical formula can be expressed as (C_3_H_3_N)_n_. Therefore, a small amount of N element can be found in the element mappings and EDS results after carbonization of the polyacrylonitrile precursor at a high temperature. Other similar work using this CF has also demonstrated the presence of the N element.^[^ ^19^ ^]^ The morphologies of CF, CFH, and CFK have been characterized and exhibited in Figure S3, Supporting Information. The XRD patterns in Figure 2d reveal that the crystallinity of the samples has not been destroyed during the preparation process. The change in the intensity of the diffraction peak at 20°–30° indicates that the (001) plane of the material is damaged to different degrees during the treatment process, leading to a reduction in the diffraction intensity.^[^ ^30^ ^]^ Specific surface area and the pore volume of the four samples have been analyzed and the results are demonstrated in Figures 2e,f. The specific surface area of CF has been improved from 4.4 m^2^ g^−1^ to 34.9 m^2^ g^−1^ after the initial annealing treatment, as shown in Figure S4a, Supporting Information. The etching efficiency and depth can be upgraded by the improvement of specific surface area. CFK without first annealing treatment exhibits lower specific surface area and pore volume than CFHK, which indicates that the two‐step treatment of the CF is beneficial to enhancing the contact area between the material and the electrolyte, thus boosting the electrochemical performance for supercapacitors. Finally, CFHK with the highest specific surface area of 1124.4 m^2^ g^−1^ and a pole volume of 0.9213 cm^3^ g^−1^ has been obtained. The large pore volume and specific surface area can provide abundant active sites for the adsorption and desorption of the charges, thereby exhibiting outstanding energy storage performance. Figure S4b, Supporting Information reflects the Raman results of the four samples with almost the same ratios of *I*_D_: *I*_G_, which reveal that the annealing and the introduction of KOH do not change the number of defects evidently.^[^ ^31^, ^32^ ^]^ The four samples have been characterized by FTIR and the result is demonstrated in Figure S4c, Supporting Information. The wide and weak bands between 1330 and 940 cm^−1^ can be attributed to the response of C–O stretching vibration. The bands from 1490 to 1740 cm^−1^ are ascribed to C≐O stretching, C≐C stretching and O–H bending modes. The strong and wide bands at 3420 cm^−1^ can be attributed to the O‐H stretching vibrations of hydroxyl and absorbed water molecules in as‐obtained materials.^[^ ^33^, ^34^ ^]^ The XPS results of four samples have been demonstrated and discussed in Figure S5, Supporting Information.

**Figure 3:**
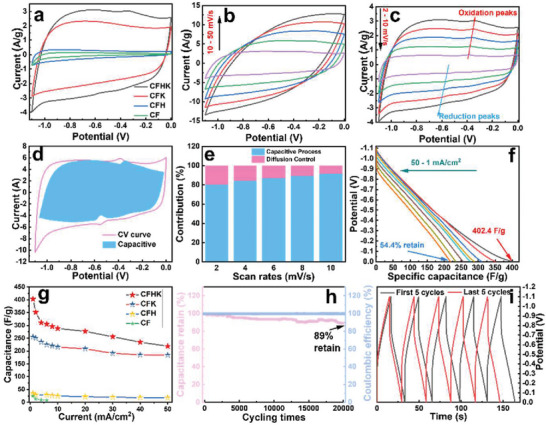
Electrochemical performance of as‐prepared samples. a) CV curves of CF, CFH, CFK, and CFHK. b,c) CV response curves of CFHK under different scan rates. d,e) The capacitance contribution in capacitive and diffusion control process. f) The discharge curves of CFHK. g) The specific capacitance of four samples under different current densities. h) Cyclic performance and the Coulombic efficiency of CFHK. i) The galvanostatic charge‐discharge curves before and after 20 000 cycles.

The electrochemical performance of the samples has been first evaluated in a typical three‐electrode system and the results are present in **Figure** **3** and Figures S6–S8, Supporting Information. The CV response curves of the four materials with rectangular‐like shapes at the scan rate of 10 mV s^−1^ in Figure 3a reflect the typical capacitive behavior. Among them, CFHK exhibits the largest area with the best current response and capacitance performance. Figure 3b delivers the CV curves of CFHK under different scan rates from 10 to 50 mV s^−1^ with obvious electric double‐layer capacitance response characteristics. While a pair of redox peaks are observed at the scan rates below 10 mV s^−1^ due to the interaction between the functional groups on the surface of the material and the electrolyte, as shown in Figure 3c. According to the previous work, the storage of charge is composed of the capacitive and diffusion control processes.^[^ ^35^ ^]^ The contributions of those two processes for CFHK at different scan rates have been calculated and the corresponding results are displayed in Figures 3d,e. The surface‐controlled capacitive contribution is 80.4% at the scan rate of 2 mV s^−1^ and delivers 84.4%, 86.8%, 89.12%, and 91.6% at the scan rates of 4, 6, 8, and 10 mV s^−1^, respectively. Surface control has a faster ion response compared with diffusion control, which is beneficial to the rate ability of the electrode. Figure 3f discloses the discharge curves of CFHK with a linearity feature, revealing a typical electrostatic adsorption behavior for the electrode. The specific capacitance of the material has been calculated and the results are expressed in Figure 3g. As expected, CFHK delivers the highest specific capacitance of 402.4 F g^−1^ at 1 mA cm^−2^ and maintains 218.8 F g^−1^ at 50 mA cm^−2^. Cyclic stability is also a key feature of supercapacitor materials. In Figure 3h, 89% capacitance retention after 20 000 cycles has been achieved, proving the outstanding cyclic stability of the CFHK electrode. The relative galvanostatic charge‐discharge (GCD) curves of CFHK before and after 20 000 cycles without significant charges in Figure 3i also confirm its outstanding cyclic performance. The excellent cyclic stability of CFHK ensures long‐lasting energy storage and conversion, which is supposed to be an ideal material for a long‐term maintenance‐free energy storage device.

**Figure 4:**
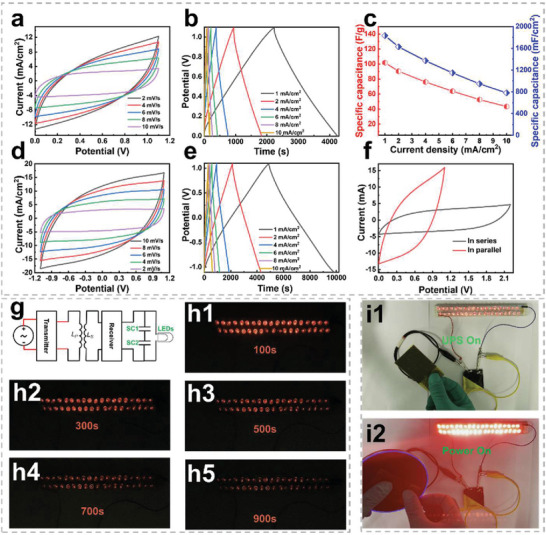
The electrochemical performance and the applications of CSSC. a) CV response, b) GCD curves under the potential window of 0–1.1 V. c) The specific capacitance of CSSC. d) CV response curves, e) GCD curves under the potential window of −1.1–1.1 V. f) CV curves of two CSSC in series and parallel. g) Schematic diagram of using CSSC as an energy storage device to light 38 LEDs by fast wireless charging. h1‐h5) Brightness graph of 38 LEDs over different times powered by CSSC. i1‐i2) The practical performance of CSSC acts as an uninterrupted power supply (UPS).

To further verify the practical application, CF‐based symmetric, asymmetric, and CSSC have been prepared and evaluated, and the relative results are exhibited and discussed in Figures S9‐S14, Supporting Information, and **Figure** **4**. The CV response curves with distinct capacitive characteristics are revealed in Figure 4a. Figure 4b illustrates the GCD curves of the CSSC. The GCD curves with isosceles‐triangle shapes imply ideal capacitive behavior, which agrees with the CV curves. According to the GCD curves, the CSSC delivers a maximal specific area capacitance of 1831.8 mF cm^−2^ and a specific mass capacitance of 101.7 F g^−1^, as shown in Figure 4c. In addition, when the voltage is extended to −1.1–1.1 V, the CV response and GCD curves of the CSSC still exhibit the typical capacitive characteristic, as revealed in Figures 4d,e. To verify the practical application prospects of supercapacitors, two CSSCs have been assembled and the CV response curves under parallel and series conditions were tested. The corresponding results are shown in Figure 4f. When two CSSCs are connected in series, the operating voltage of the device can be effectively extended to 2.2 V with ideal capacitive performance. Based on this, a wireless charging device has been designed to verify the application prospects of supercapacitors. Figure 4g exhibits the circuit diagram of the wireless charging device. The external commercial wireless charging board generates an alternating magnetic field through the wireless charging transmitter, and the receiver is affected by the alternating magnetic field to generate an induced electromotive force and an induced current. The induced current through the circuit transports electrical energy into CSSC for storage. As the result, 38 LEDs in parallel can be lightened for >900 s by two CSSC in series after a wireless charging time of ≈2 s, as shown in Figures 4h1‐h5 and Movie S2, Supporting Information. In addition, the CSSC can also be used as a UPS device. As exhibited in Figures 4i1, i2 and Movie S3, Supporting Information, when the external power supply is connected, part of the electrical energy is used to power the loaded LEDs, while the others are stored in CSSC. The LEDs will be supplied with CSSC as the UPS immediately when the external power supply is removed.

### 2.3 Comprehensive Characterization and Discussion of Continuous Self‐Power System

**Figure 5:**
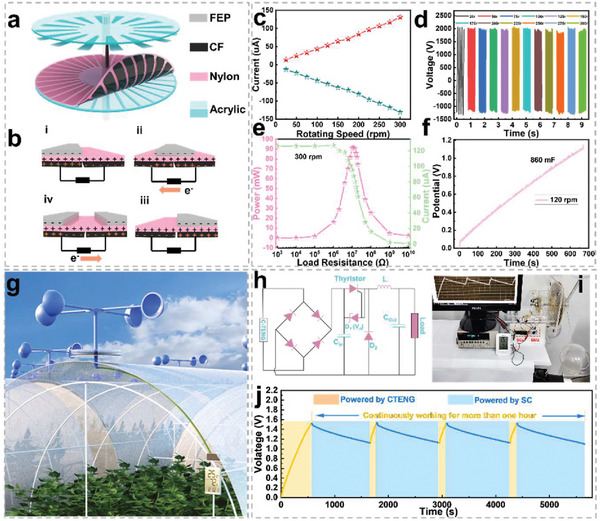
Schematic and output performance of CECIS. a) Schematic diagram about the structure of C‐TENG for the energy harvesting part. b) Working principle of the C‐TENG. c) Short circuit current. d) Peak‐to‐peak voltage of C‐TENG under different rotating speeds. e) The output power and matching impedance of C‐TENG. f) Voltage‐time curves of the CSSC charged by C‐TENG under 120 rpm. g) Blueprint of the CECIS to drive the hygrothermograph. h) Circuit schematic diagram of the CECIS. i) Photograph of the CECIS to drive the hygrothermograph. j) the voltage curves of CECIS when it is worked by the wind to drive the hygrothermograph for continuous power supply.

C‐TENG has been designed as an energy collection part for CECIS. **Figure** **5**a reveals the 3D structure of the C‐TENG with the disk‐shaped stator and a rotor. Specifically, the stator is composed of an acrylic disc, acrylic skeleton, nylon film, and CF. Due to the distinguished electrical conductivity and flexibility of CF, it not only can be served as a buffer layer to support friction materials but also as the induction layer and current collector to simply the structure of the C‐TENG. The FEP film is assembled by double‐sided adhesive on another acrylic disc as a rotor. The rotor relates to the rotating shaft through a coupling, and friction occurs between the nylon and the FEP on the rotor under the action of rotation. Figure 5b shows the working diagram of C‐TENG, which is a typical freestanding model with four stages. In the initial state, the different electronegativity of the nylon and FEP films will result in charge transfer upon contact and induction of charge on the sensing layer, as demonstrated in Figure 5bi. When the rotor starts to move, the positive charge accumulated in the sensing layer flows to the other electrode through the external circuit, as shown in Figure 5bii. When moving to the next stage, in Figure 5biii, the charge distribution between the two electrodes is opposite to the initial state. Ultimately, sustained friction can drive the periodic transfer of electrons between the sensing layers through an external load, thus resulting in periodic currents in the external circuit.^[^ ^27^ ^]^ The output performance of C‐TENG has been characterized. As a result, C‐TENG delivers a peak current of ≈10 uA at 25 r and increases up to 130 uA at 300 r, as shown in Figure 5c. It is worth noting that the linear relationship between the C‐TENG output current and rotational speed without obvious attenuation indicates that C‐TENG has a stable rising current output with the increase of rotational speed. Figure 5d shows the peak‐to‐peak voltage output curve from 25 r to 300 r. The peak‐to‐peak voltage of C‐TENG is stabilized at ≈3150 V in the rotating speed from 25 r to 300 r. The positive voltage is much higher than the negative voltage, the reasons for this have been explained in Figure S15, Supporting Information. In addition, the maximal output power of C‐TENG reaches 91.5 mW when the external matching impedance is 1.2 × 10^7^ Ω, as shown in Figure 5e. Figure S16, Supporting Information, depicts the transferred charges of C‐TENG, which delivers >750 nC under 25 rpm and maintains 640 nC at 300 rpm. The good performance of C‐TENG can meet the needs of high‐power output. As shown in Figure 5f, a supercapacitor (860 mF, assembled by CFHK) has been charged up to 1.1 V within 700 s by C‐TENG under 120 rpm, showing well‐matched characteristics between energy harvesting and storage. The cycle life of C‐TENG is also one of the important factors determining the cycle stability of the whole CECIS. As a result, the C‐TENG is tested for over 100 000 cycles at 75 rpm. During the whole process, the peak short‐circuits current output of C‐TENG is always ≈30 µA and its output curve is very stable except for a small fluctuation in the middle of the test. which are demonstrated and discussed in Figure S17, Supporting Information.

Thanks to the excellent performance of CSSC and C‐TENG, CECIS has been assembled by C‐TENG, CSSC, and the management circuit to achieve a continuous self‐powered system integrating energy collection and storage. To simulate the real energy harvesting scene, C‐TENG is driven by the wind cup to collect energy under the wind, as depicted in Figure 5g and Movie S4, Supporting Information. Figure 5h demonstrates the circuit diagram of CECIS. In the process of charging the supercapacitors by C‐TENG, the extremely high peak‐to‐peak voltage can be converted into a safe and reasonable voltage by the power management circuit, and the specific conversion process is shown in Figure S18, Supporting Information.^[^ ^36^, ^37^ ^]^ AC from C‐TENG is converted into DC through the management circuit, part of which is supplied to the external load, and the excess is stored by the CSSC in parallel. When the C‐TENG stops working, the electric energy stored in the CSSC can be supplied to the external load continually, thus realizing the uninterrupted operation of the external load throughout the whole process. Figure 5i shows the actual application scenario, and the corresponding complete voltage curve is shown in Figure 5j. The system charges a CSSC (128.3 mF, Figure S19, Supporting Information) to 1.5 V within 10 min driven by a wind under the speed of ≈3 m s^−1^. A hygrothermograph (≈0.18 mW) is powered for more than 18 min by CSSC when the wind stops. After 18 min, the voltage of the CSSC reduces to 1.1 V with the fade of the hygrothermograph. Driven by the wind, the CSSC is recharged from 1.1 V to 1.5 V within 2 min by C‐TENG under the wind speed of 3 m s^−1^. It is worth noting that the work of the hygrothermograph never stopped during the whole process of the application even though sometimes the C‐TENG does not work because there is no wind energy to provide. Benefiting from the superior energy storage performance of the supercapacitor and outstanding output characteristics of C‐TENG, the ratio of *t*_c_:*t*_d_ is determined to be 9.6:1. It means that in practical application scenarios, CECIS can achieve a 24‐h continuous self‐power supply as long as the actual working time exceeds one‐tenth of a day.

## 3 Conclusions

In summary, CECIS has been designed and assembled by C‐TENG and CSSC to achieve the collection, conversion, and storage of high‐entropy energy for a continuous self‐power system. As the active electrode materials for CSSC, the CFHK obtained after the simple treated CF not only exhibits a maximal capacitance of 402.4 F g^−1^, but also reveals ideal capacitance performance in symmetric, asymmetric, and solid‐state supercapacitors. The packaged CSSC with fast charging and slow discharging characteristics ensures 38 LEDs lighten for more than 900 s after 2 s of wireless charging. Besides, the original CF has also been applied in C‐TENG for energy harvesting, and simultaneously serves as the buffer layer, sensing layer, and current collector of the device. The assembled C‐TENG exhibits outstanding output performance with a maximal output power of 91.5 mW, and a CSSC (860 mF) has been successfully charged to 1.1 V under the rotating speed of 120 rpm. Benefiting from the superior performance of CSSC and C‐TENG, those two units have been matched through circuit management to finally achieve the CECIS with excellent continuous output characteristics. As a result, CSSC successfully drives a hygrothermograph for >18 min after charging with C‐TENG for nearly 2 min. The ratio of *t*_c_:*t*_d_ is 9.6:1, which means that the whole system only needs one‐tenth of the effective working time to realize the all‐weather continuous self‐power supply. The outstanding performance of the CECIS will have broad development prospects and practical significance for the collection, storage, and supply of high‐entropy energy.

## Conflict of Interest

The authors declare no conflict of interest.

## Author Contributions

P.J. and Q.L. contributed equally to this work. P. Ji: conceptualization, methodology, measurement, visualization, data curation, formal analysis, writing – original draft; Q. Li: data curation, conceptualization; X. Zhang, Y. Hu, X. Han: investigation; D. Zhang: visualization; C. Hu: conceptualization, Y. Xi: conceptualization, resources, supervision, writing – review & editing.

## Supporting information

Supporting information

Supplemental Movie 1

Supplemental Movie 2

Supplemental Movie 3

Supplemental Movie 4

## Data Availability

The data that support the findings of this study are available in the supplementary material of this article.
