# Supplementary material for: Achieving Continuous Self‐Powered Energy Conversion‐Storage‐Supply Integrated System Based on Carbon Felt
Source: Adv Sci (Weinh). 2023 Mar 6;10(13):2207033. doi: 10.1002/advs.202207033 (PMC10161012; doi:10.1002/advs.202207033)
Supplement: Supplementary file 1 — Supporting information [file ADVS-10-2207033-s005.pdf]

ADVANCED SCIENCE
Open Access

Supporting Information

for *Adv. Sci.*, DOI 10.1002/advs.202207033

Achieving Continuous Self-Powered Energy Conversion-Storage-Supply Integrated System Based on Carbon Felt

*Ji Peiyuan, Li Qianying, Zhang Xuemei, Hu Yawen, Han Xiangyu, Zhang Dazhi, Hu Chenguo and Xi Yi**

Supporting Information

Achieving continuous self-powered energy conversion-storage-supply integrated system based on carbon felt

*Ji Peiyuan, Li Qianying, Zhang Xuemei, Hu Yawen, Han Xiangyu, Zhang Dazhi, Hu Chenguo, Xi Yi**

Supplementary Equations

$$C = I\Delta t/m\Delta V \quad (1)$$

$$E = 1/2C\Delta V^2/3.6 \quad (2)$$

$$P = E/\Delta t \quad (3)$$

Where C(F g^{-1}) is the specific capacitance, E(Wh Kg^{-1}) is the specific power, and P (W Kg^{-1}) is the specific energy. I, Δt, m, and ΔV is the discharge current, discharge time, total mass of the active material in the electrode, and voltage change within the discharge time, respectively.

Experimental

Nickel foam (NF), FEP film, Nylon film, and acrylic board were bought from Taobao, Carbon felt (CF) was bought from Jinglong special carbon. PVA-1788 and

KOH were purchased from Aladdin. All the reagents were directly used without further purification.

Preparation of electrode materials

The schematic diagram of the main principles of synthesis is shown in **Figure S1 (ESI)**. In a typical synthesis process, CF was firstly annealed at 800 ^{o}C in a tube furnace for 5 h under flowed Ar. After cooling down to room temperature, the CF was taken out and named CFH. The as-obtained CFH was immersed into 1 M KOH solution for 12 h followed by drying at 100 ^{o}C for another 12 h. The sample was then transferred to a tube furnace and annealed at 800 ^{o}C for 5 h under flowed Ar. After that, the sample was washed with deionized water and dried in a vacuum oven night and named CFHK. The synthesis method of CFK is like the CFHK except for the first annealing process. The final area mass is 23, 19.8, and 8.86 mg cm^{-2} for CFH, CFK, and CFHK, respectively.

To prepare the positive electrode material, NF was used as the substrate, and the deposition mass was controlled by electrodeposition time to obtain the positive electrode material that could well match the capacitance of the CFHK. Specifically, NF was treated with 1 M hydrochloric acid, deionized water, and absolute ethanol sequentially and then dried in a vacuum drying oven. 2 mM $Ni(NO_3)_2$ and 2 mM $Co(NO_3)_2$ were dissolved in DI water to form the electrodeposition solution. The treated NF were immersed in the above solution. Using the chronopotentiometry method, NF, Pt, and Ag/AgCl electrodes were used as the working electrode, the counter electrode, and the reference electrode, respectively. The deposition time was finally determined to be 1200 s, and the voltage was set as -1.1 V. The obtained cathode material was taken out, rinsed with deionized water and alcohol, and dried at 60 ^{o}C overnight. The final mass loading on the NF was 7 mg cm^{-2}.

Preparation of solid-state electrolyte and organic membrane

PVA (6 g) was slowly added into a 100 ml beaker with 45 ml of DI water and stirred vigorously at 85 ^{o}C for 6 h. After that, 10 ml 6 M KOH solution was added and stirred at 70 ^{o}C for 2 h to obtain a clear PVA-KOH organic electrolyte.

To obtain the organic membrane, the prepared organic electrolyte was evenly spread on the acrylic mold, put in the refrigerator, and frozen at -103 ^{o}C for 12 h followed by being taken out and placed at room temperature for 1 h. The above process was repeated 3 times to obtain a well-polymerized PVA-KOH organic membrane. Thereafter, the membrane was cut into the required size and immersed in a 6 M KOH solution for use.

Assembly of CF-based solid-state supercapacitors

The as-prepared CFHK was immersed in fresh PVA-KOH organic electrolyte at 70 °C to acquire good contact between the material and the organic electrolyte. It was then attached to both sides of the PVA-KOH organic membrane, sealed with tape to isolate the air, and placed for 24 h before evaluating the capacitance performance.

Fabrication of CF-based TENG device

CF-based TENG (CTENG) consisted of a rotor and stator. Nylon films and FEP films (thickness of 0.03mm) were used as friction materials. The rotor was composed of an acrylic substrate and FEP film. Acrylic (thickness of 4mm) was cut into a hollow shape disk with an inner diameter of 10 mm and an outer diameter of 115 mm by a laser cutting machine. FEP film was cut into 16 fan shape pieces, which were attached to one side of the acrylic board, and then the rotor was formed by connecting the stainless-steel shaft and the other side of the acrylic board through a coupling flange. For the stator, the acrylic board was cut into the same hollow circle with an inner diameter of 10 mm and an outer diameter of 115 mm. A 2 mm thick acrylic plate was cut into a hollow circle containing 32 fan-shaped holes as a support skeleton by a laser

cutting machine. CF was precisely cut into a fan shape under the laser and filled in the skeleton as the buffer layer, the induction layer, and the conductive layer of the friction material simultaneously. A whole nylon film with an inner diameter of 10 mm and an outer diameter of 115 mm was pasted on the CF with the captain tape as a positive friction material.

Structure characterization

The morphology and the microstructure of the CF were characterized by Field Emission Scanning Electron Microscopy (FESEM, Tescan Mira 3, Czech). A transmission Electron Microscope (TEM, Thermo scientific, Talos F200S, Czech) was applied to investigate the lattice information and distribution of the elements. The X-Ray Diffraction (XRD) results were acquired by PANalytical X'Pert with Cu Kα radiation. The specific surface area and pore volume of the samples were characterized by a Fully automatic multistation-specific surface and aperture analyzer (Quadrasorb 2MP, Quantachrome Instruments, USA). The defects and disordered degree of materials can be characterized by Raman Spectroscopy (LabRAM HR Evolution HORIBA, Jobin Yvon S.A.S. France). The types of functional groups in the sample and their chemical environment are characterized by the Fourier Transform Infrared Spectrometer (FTIR, Nicolet iS50, Thermo Fisher Scientific, USA).

Electrochemical measurements

The electrochemical performance of as-prepared samples was evaluated by an electrochemical workstation (CHI660E, Chenhua, Shanghai). Cyclic voltammetry (CV), galvanostatic charge-discharge (GCD), and electrochemical impedance spectroscopy (EIS, 0.1-100 kHz) were measured by a typical three-electrode system in 6 M KOH. Pt plate, active materials, and Hg/HgO electrode were used as the counter, active, and reference electrodes respectively. The capacitance behavior of symmetric, asymmetric, and solid-state supercapacitors was evaluated by CV and GCD methods.

The specific capacitance, power, and energy were calculated by equations 1-3 in supporting information.

For the wireless charging part, when charging the solid-state devices, the purchased commercial wireless charging board is connected to the power supply as the transmission port of electric energy. The positive and negative poles of the assembled solid-state supercapacitor are connected to the output end of a wireless receiving coil pasted on one side of the solid-state supercapacitor. After charging, the positive and negative electrodes of the solid-state capacitor are connected to the LED lamp to light the LED.

Electrical measurement of the C-TENG

The short-circuit current (I_{SC}), short-circuit charge transfer (Q_{SC}), and matching the impedance of the device was measured by an electrometer (Keithley 6517, Tektronix). Open-circuit voltage (V_{OC}) was evaluated by 370. A motor was connected to the shaft of the rotor to drive the device.

Characterizations of continuous self-power system

A wind cup was attached to the rotor shaft to collect wind energy. An adjustable fan was used in the self-powered system to accurately simulate natural wind. Specifically, the wind-driven wind cup drives the rotor at the other end to rotate, and the current generated by the mutual friction with the stator is processed by the rectifier circuit and then convert to the supercapacitor in parallel for storage. After reaching the working voltage, it drives the temperature and humidity meter to work. When the voltage drops to the cut-off working voltage of the thermometer, start the fan again to drive the entire device to run. Keithley 6514 was used in parallel across the supercapacitor to detect the voltage versus time throughout the operation.

Supplementary Figures

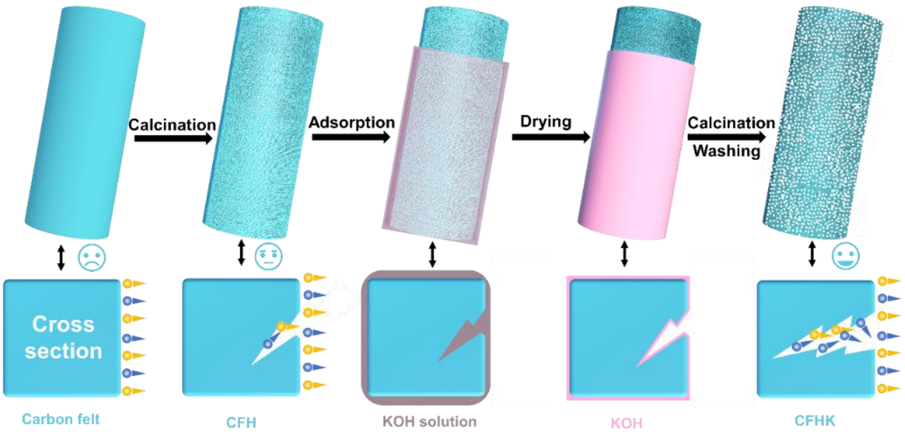

Figure S1 Schematic synthesis process of CFHK.

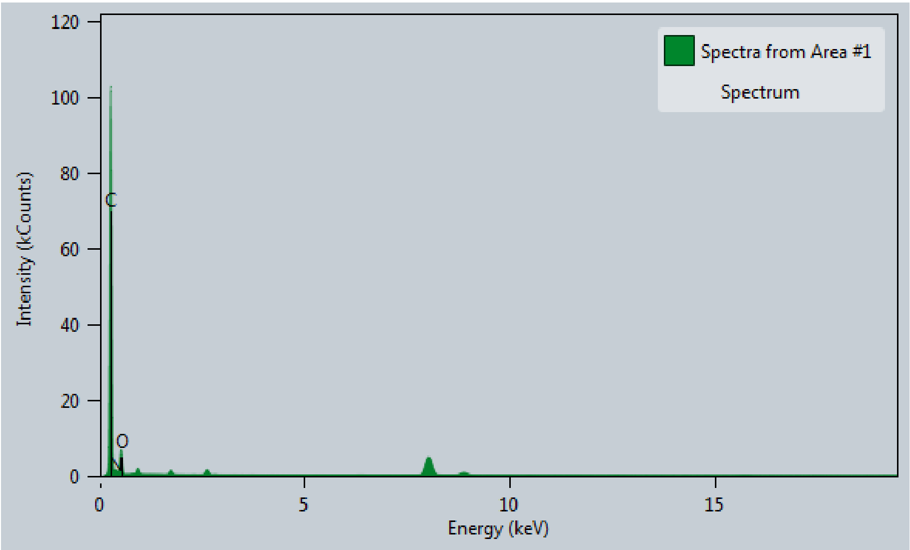

Figure S2 EDS spectrum of CFHK.

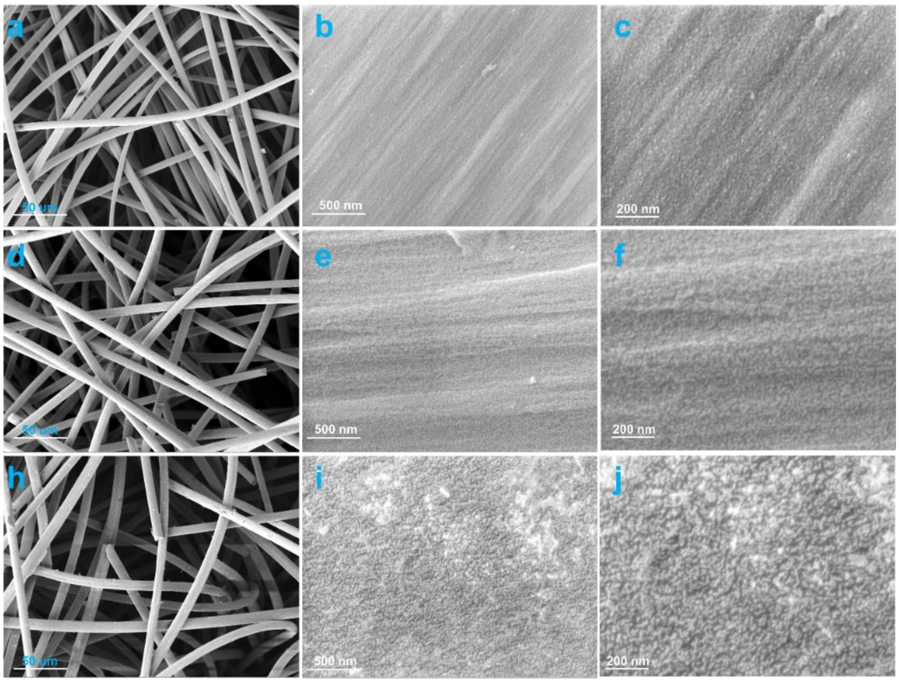

Figure S3 SEM images of CF (a-c) CFH (d-f) and CFK (h-j).

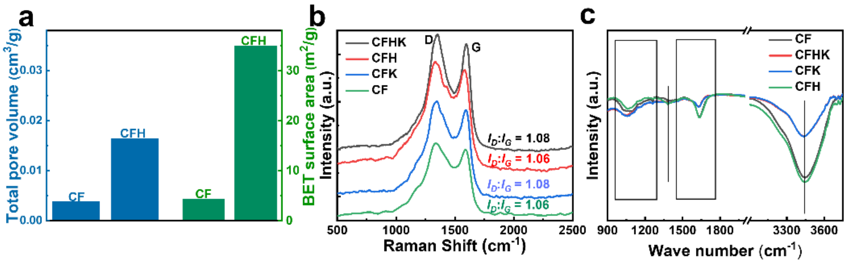

Figure S4 Pore volume and surface area of CF and CFH(a, b). Raman spectrum of CF, CFH, CFK, and CFHK (b). Fourier infrared transform spectrum characterization results of the four samples (c).

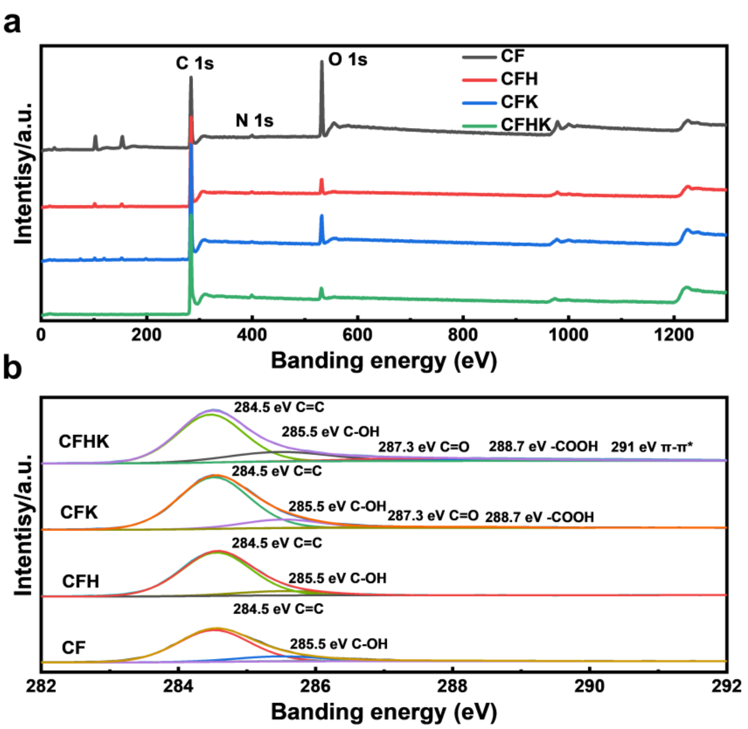

Figure S5 XPS results of four samples.

Figure S5 (ESI) demonstrates the XPS results of four samples. As expected, the characterized peaks of C, N, and O can be observed in Figure S5a (ESI). Figure S5b (ESI) exhibits the banding energy results of C1s. Compared with the initial CF, the reduced C-OH content of CFH indicates that oxygen tends to be released from the material under high temperatures.[1] After KOH treatment, newly formed C-OH and C-OOH bonds indicate that the method of KOH treatment of the material is effective. The C-OH bond is easily broken as the temperature increases, resulting in a corresponding decrease in the strength of the C-OH bond.[2] At the same time, the C=O bond is formed because of the recombination of these atoms. As for CFHK, a more obvious peak appears around 291 eV, which can be summarized as π-π*. The existence of π-π* indicates the delocalized electrons such as aromatic rings between graphite.[3] Those results agree with the SEM and TEM images in Figure 2a-c, there are many mesopores on the surface of CFHK, and ultrathin carbon shells like graphene have been formed between these pores, thus resulting in the delocalization of the electrons.[4]

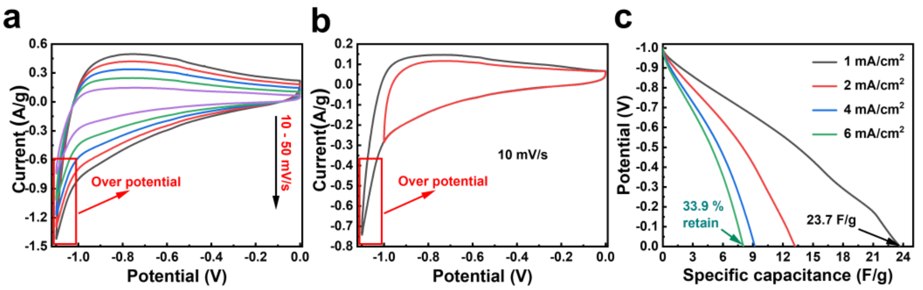

Figure S6 Electrochemical performance of CF without treatment. CV response under different scan rates (a, b). the discharge curves under different current densities (c).

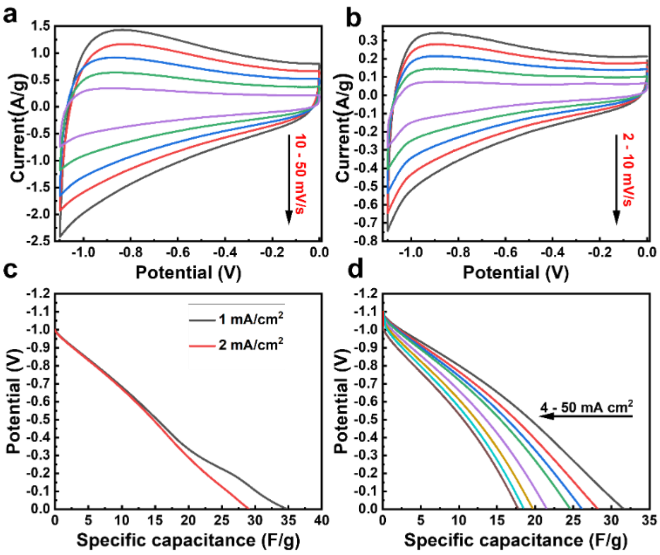

Figure S7 Electrochemical performance of CFH. CV response under different scan rates (a, b). the discharge curves under different current densities (c, d).

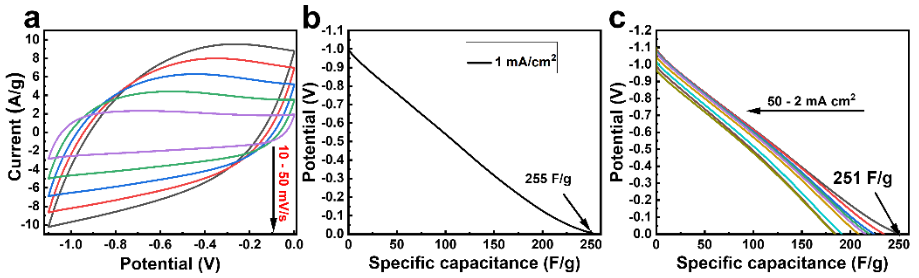

Figure S8 Electrochemical performance of CFK. CV response under different scan rates(a). the discharge curves under the potential of -1 V – 0 V (b) and -1.1 V- 0 V (c).

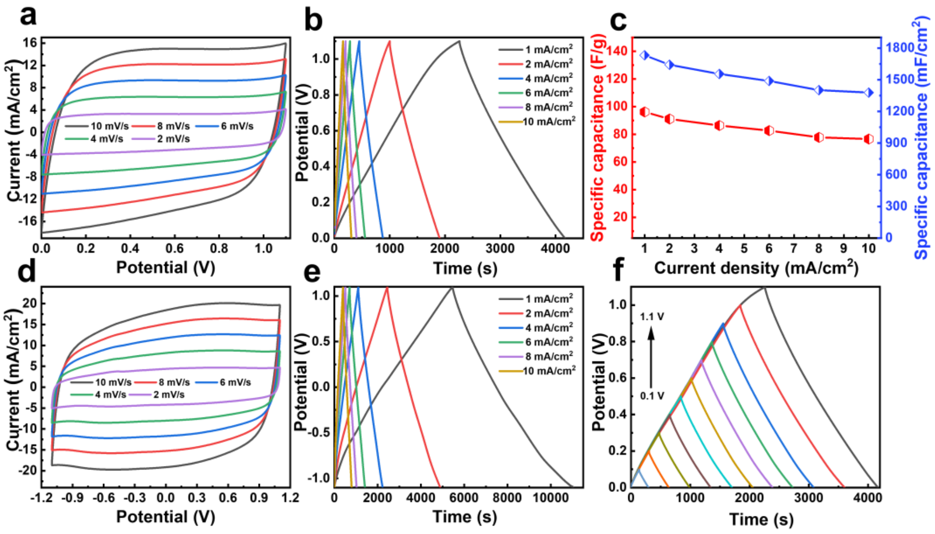

Figure S9 Electrochemical performance of symmetric supercapacitors in 6 M KOH. CV response curves under the potential window of 0 - 1.1 V (a). GCD curves under different current densities (b). The specific capacitance of symmetric supercapacitors (c). CV response curves under the potential window of -1.1 V - 1.1 V (d). GCD curves under the potential window of -1.1 V - 1.1 V (e). GCD curves under the different potential windows of 0 - 1.1 V (f).

For the assembled symmetric supercapacitor, the theoretical maximum capacitance has the following relationship with the single-electrode capacitance,

$$\frac{1}{C} = \frac{1}{Ca} + \frac{1}{Cc}$$

Ca and Cc are the capacitance of the anode and cathode, respectively. Based on the above equation, for a symmetric supercapacitor, the total capacitance is about 1/4 of a single electrode. In the typical measurement in liquid conditions, the maximum capacitance is 98.01 F g^{-1}, which is comparable to the theoretical maximum capacitance. At the same time, for symmetrical supercapacitors, ideal capacitance characteristics are also exhibited under the test voltage of -1.1-1.1V, and an ideal isosceles triangle is exhibited at any voltage range.

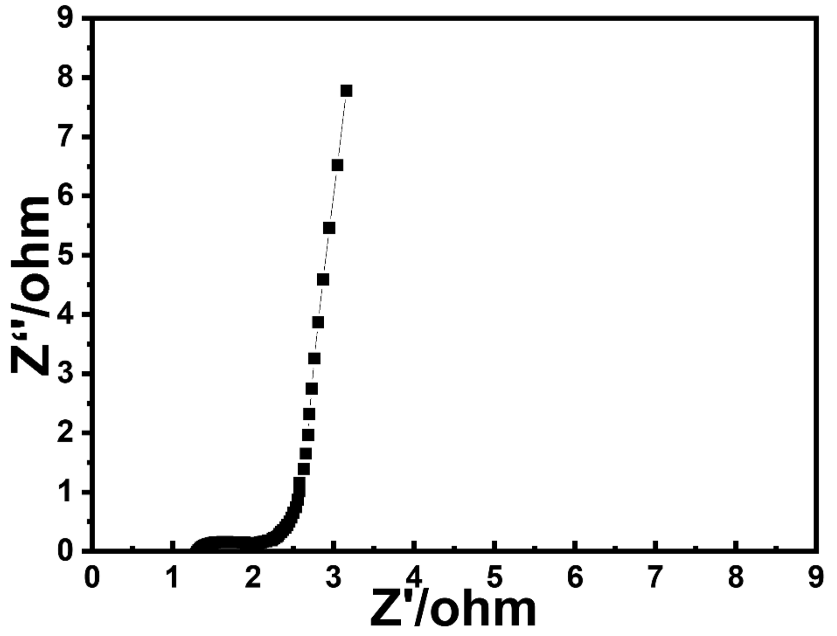

Figure S10 EIS result of the symmetric supercapacitor.

Figure S10 reveals the EIS result of the symmetric supercapacitor. Thanks to the superior conductivity and hydrophilicity of the CF, the equivalent series resistance(R_s) of the symmetric device is only 1.3 Ω. Moreover, a nearly vertical straight line can be observed in the low-frequency region, indicating the ideal capacitive feature.

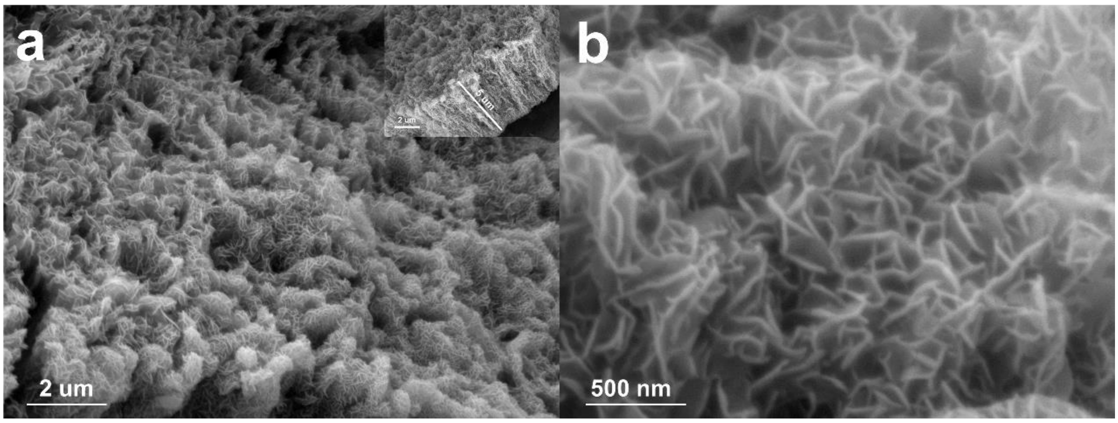

Figure S11 SEM images of NiCo composites in different magnifications.

The morphology of the material is shown in the figure above, showing a uniformly distributed nanostructured shape. This nanostructure has good stability and abundant

active sites, providing a rich number of charges through the redox reaction with the alkaline electrolyte.

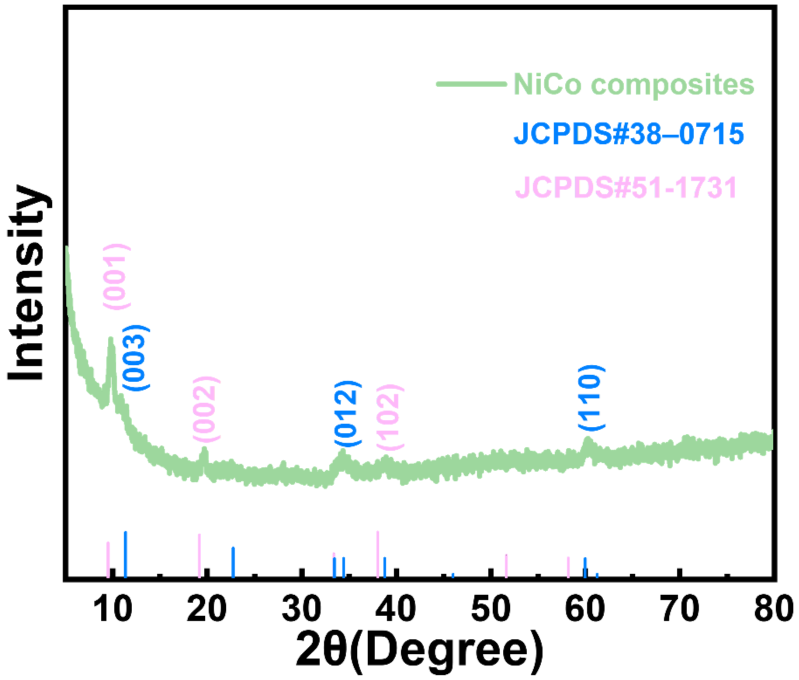

Figure S12 XRD patterns of NiCo composites.

Since the diffraction peaks of the NF are too strong, we collected the powder material under ultrasound and characterized it by XRD, as shown in Figure S12. Among them, the peaks at 9.5°, 19.2°, and 38° can be attributed to the (001), (002), and (102) planes of $Co(OH)_2$, while the remaining peaks at 11.3°, 33.5°, and 60° are well matched to the (003), (012), and (110) planes of $Ni(OH)_2 \cdot 0.75H_2O$.[5-7] No other impurity peaks can be observed.

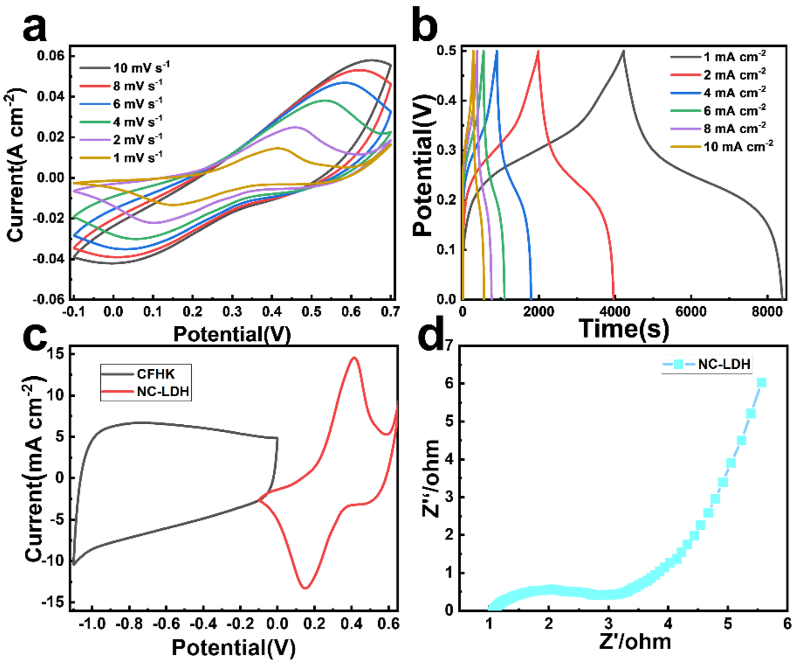

Figure S13 Electrochemical performance of the positive materials**.** The CV response curves (a) and GCD curves (b). CV response curves of the positive and negative electrodes under different potential windows (c). EIS results (d).

To explore the practicality of carbon felt as the negative electrode material, we chose an easily prepared nickel-cobalt double hydroxide as a positive electrode material to assemble an asymmetric supercapacitor. It can be found from the CV and GCD curves that the material not only exhibits obvious pseudocapacitance characteristics but also delivers a comparable capacitance to that of the negative electrode material at the same current density. EIS analysis shows that this material has a small charge transfer resistance, so that is can act as a suitable material for asymmetric supercapacitors.

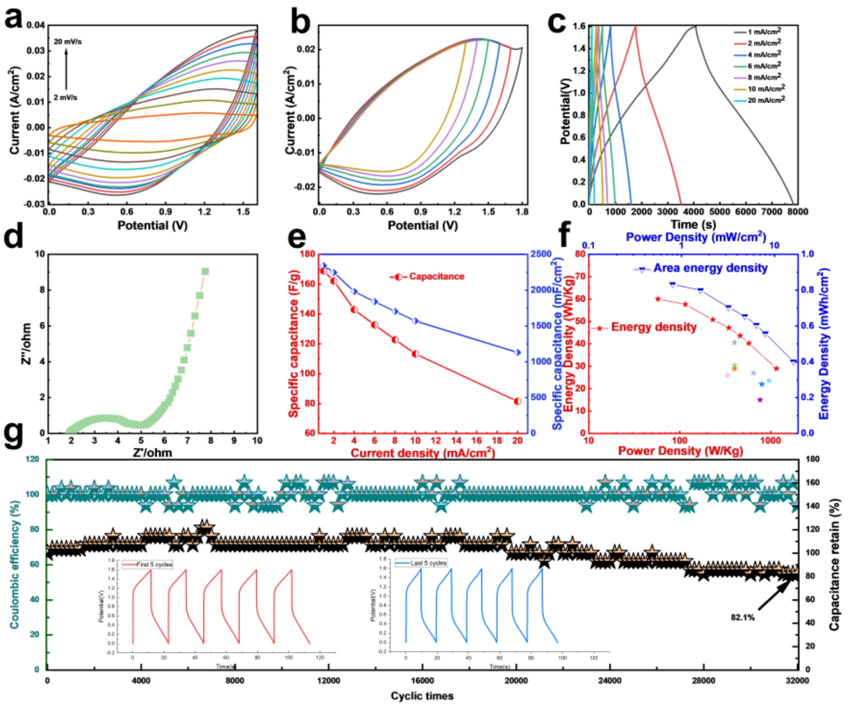

Figure S14 Electrochemical performance of asymmetric supercapacitors(ASC). CV curves (a, b), GCD curves (c), EIS results (d), the specific capacity performance (e), energy density and power density compared with the other similar works (f),[8-15] and cyclic performance of the ASC (g), the first and last five GCD curves in 32000 cycles (inset).

In the performance test of the assembled asymmetric device, due to the good capacity matching between the positive and negative electrodes, the CV has a quasi-rectangular shape, and the actual GCD curve shows an approximate isosceles triangle shape. At the same time, the fabricated asymmetric capacitor also has a small charge transfer resistance. The assembled asymmetric supercapacitor not only exhibits a specific mass capacitance of 168.9 F g^{-1} and an area capacitance of 2341.25 mF cm^{-2} but also delivers superior specific power and specific energy. It usually takes a long time to evaluate the cyclic stability of a device. Due to the limitation of the data storage interval of the electrochemical workstation, the record of discharge time and charge time will fluctuate. Overall, the Coulombic efficiency of the asymmetric device does not change significantly during the cycles, while the capacitance retains showed a gradual upward and downward trend. The material retains 107% of the initial capacity after 10,000 cycles and still retains 82.1 % of the initial capacity after 32,000 cycles, which proves the good stability of the device.

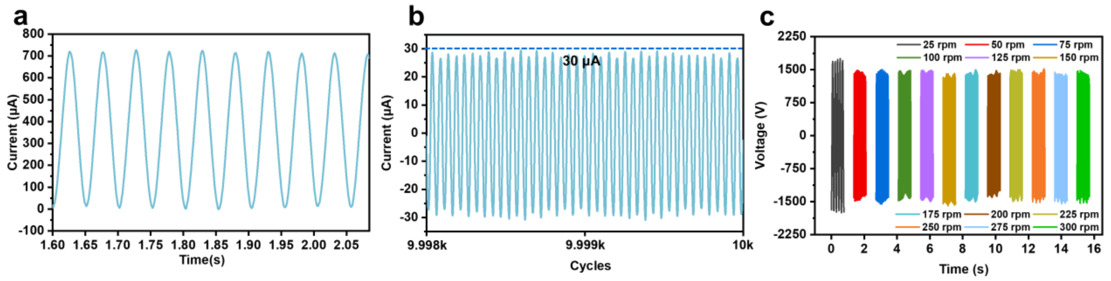

Figure S15 Basic outputs of the newly fabricated C-TENG at 75 rpm. (a) Transferred charges of C-TENG at 75 rpm. (b)Short circuit current of the newly fabricated C-TENG at 75 rpm. (b) Peak-to-peak voltage of C-TENG under different rotating speeds.

Theoretically, C-TENG, as one of the most common free-standing TENG, is symmetrical in the positive and negative parts of AC and voltage.[16] However, due to factors such as the different thicknesses of the tapes adhering the friction layer materials, the different wiring ways of the electrodes, the swing between the stator and the rotor during rotation, and the different test environments, the positive and negative parts of the C-TENG voltage have obvious asymmetry.

Therefore, a new C-TENG with the same thickness of tapes and a unified electrode connection has been fabricated. Furthermore, to reduce the swing caused by the rotation of the device, the test platform has been re-conditioned. Finally, the basic outputs of C-TENG at 75 rpm in a relatively stable environment (temperature above 22°, humidity below 70%) has been retested. In Figure S15a, b, the results show that the charge and current output of the C-TENG is 735 nC and 30 μA, respectively. However, the voltage does change. In Figure S15c, the positive and negative parts of the voltage output of C-TENG at different speeds are more symmetrical. At 25 rpm, the positive and negative voltage peaks of C-TENG are 1755 V and -1748 V, respectively. At 300 rpm, the positive and negative voltage peaks of C-TENG are 1481 V and -1534 V, respectively. The above changes reveal that the C-TENG device can indeed make the positive and negative parts of the voltage output more symmetrical. But from the above data, the peak-to-peak voltage of C-TENG does not change much, around 3000 V (the peak-to-peak voltage is around 3500 V at 25 rpm).

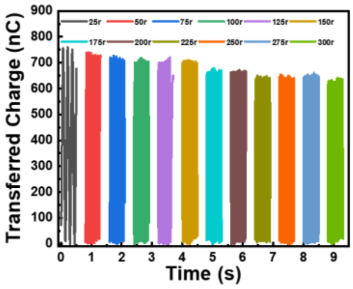

Figure S16 Transferred charges of C-TENG under different rotating speeds.

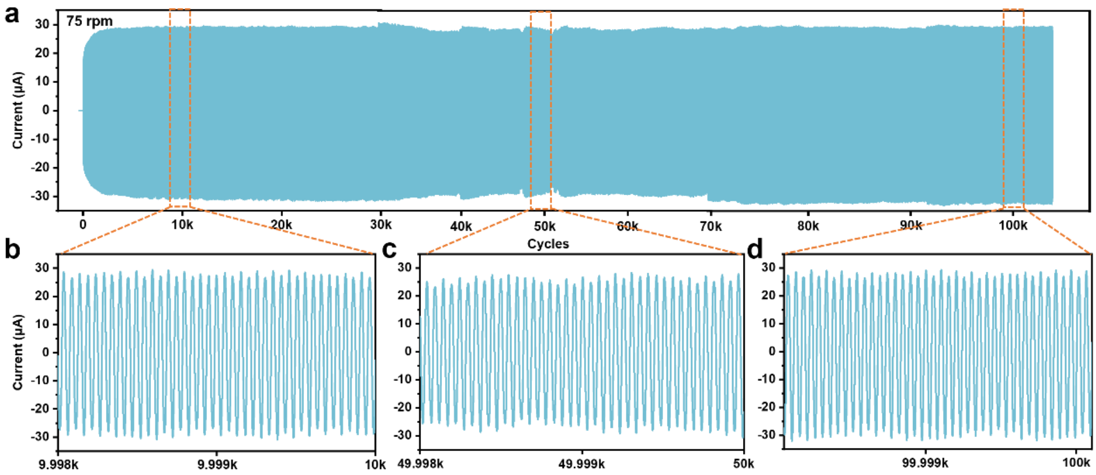

Figure S17 Stability test of the C-TENG. (a) The whole process of stability testing of C-TENG over 100,000 cycles (75 rpm). (b-c) Specific details of the current output of the C-TENG in the early, intermediate, and late stages of the stability test. It can also be seen from the figures that the current output of C-TENG has a slight decrease in the middle of the test. But after only a few cycles, the output returned to normal levels, which benefited from the softness of the carbon felt as the electrode layer and buffer layer.

To better demonstrate the cycling stability of the CECIS device, we have supplemented the stability test of the C-TENG as shown in Figure S17a. The C-TENG with 16 units was tested for over 100,000 cycles at 75 rpm. Compared to common electrode materials (copper electrodes, aluminum electrodes, etc.), carbon felt is softer. Therefore, when the carbon felt is used as the electrode of the C-TENG, it also acts as a buffer layer, which makes the C-TENG exhibit superior robustness and stability. During the whole process, the peak short-circuits current output of C-TENG was

always around 30 μA and its output curve is very stable except for a small fluctuation in the middle of the test. Figure S17b, c show the specific details of the current output of the C-TENG in the early, intermediate, and late stages of the stability test. It can also be seen from the figures that the current output of C-TENG has a slight decrease in the middle of the test. But after only a few cycles, the output returned to normal levels, which benefited from the softness of the carbon felt as the electrode layer and buffer layer.

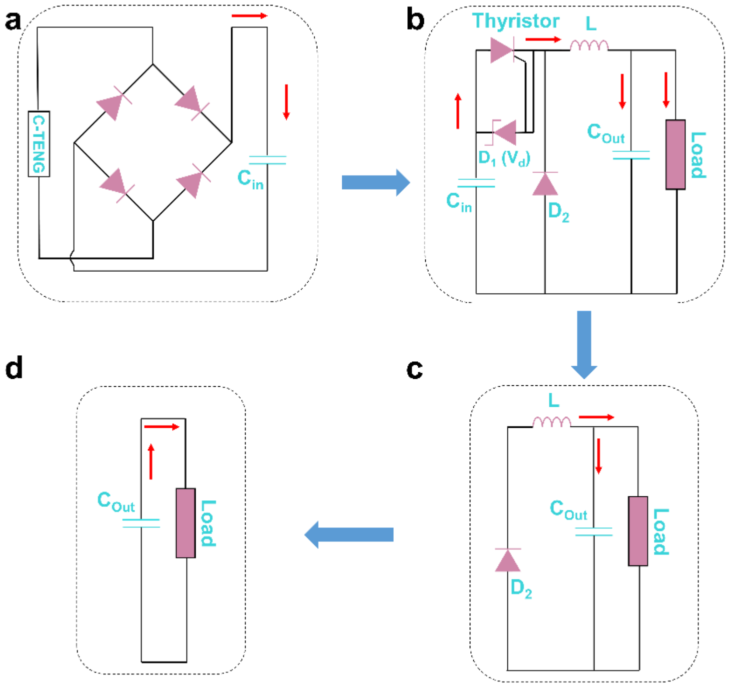

Figure S18 The energy transfer process of the energy management circuit.

The energy management circuit mainly includes three parts-a rectifier bridge, an input capacitor (C_{in}), and a step-down circuit. The thyristor, a passive electronic switch, is used in the buck circuit to reduce energy loss. When the voltage of C_{in} exceeds the breakdown voltage of the Zener diode D_1 (defined as V_d), the thyristor will be triggered by a tiny current and turns on until the voltage across it is 0. Firstly, the thyristor turns off, and the C-TENG directly charges C_{in} with the rectifier bridge (Figure S18a). After that, the energy stored in C_{in} will power the output capacitor C_{Out} and the load through the inductor L when the thyristor turns on. Simultaneously, part of the energy will be stored in the inductor (Figure S18b). Then, the energy stored in L will be supplied to

C_{Out} and the load (Figure S18c). Finally, the energy stored in C_{Out} will be transferred to the load (Figure S18d).

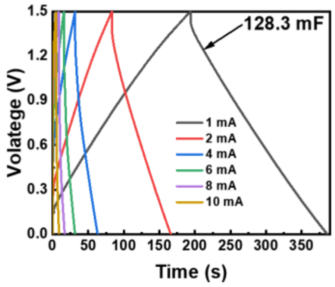

Figure S19 GCD curves of the solid-state supercapacitors applied in a continuous self-power system for driving the hygrometer.

Notes and references

[1] W. Wang, W. Liu, Y. Zeng, Y. Han, M. Yu, X. Lu, Y. Tong, *Advanced Materials* **2015**, 27, 3572-3578.

[2] Y. Li, W. Yang, W. Yang, Z. Wang, J. Rong, G. Wang, C. Xu, F. Kang, L. Dong, *Nano-Micro Letters* **2021**, 13, 95.

[3] H. Wang, J. Deng, C. Xu, Y. Chen, F. Xu, J. Wang, Y. Wang, *Energy Storage Materials* **2017**, 7, 216-221.

[4] S. He, C. Zhang, C. Du, C. Cheng, W. Chen, *Journal of Power Sources* **2019**, 434, 226701.

[5] X. Han, J. Li, J. Lu, S. Luo, J. Wan, B. Li, C. Hu, X. Cheng, *Nano Energy* **2021**, 86, 106079.

[6] X. Xuan, M. Qian, L. Han, L. Wan, Y. Li, T. Lu, L. Pan, Y. Niu, S. Gong, *Electrochimica Acta* **2019**, 321, 134710.

[7] M. U. Tahir, H. Arshad, W. Xie, X. Wang, M. Nawaz, C. Yang, X. Su, *Applied Surface Science* **2020**, 529, 147073.

[8] Y. Y. Lan, H. Y. Zhao, Y. Zong, X. H. Li, Y. Sun, J. Feng, Y. Wang, X. T. Zheng, Y. P. Du, *Nanoscale* **2018**, 10, 11775-11781.

[9] K. Zhou, W. J. Zhou, L. J. Yang, J. Lu, S. Cheng, W. J. Mai, Z. H. Tang, L. G. Li, S. W. Chen, *Advanced Functional Materials* **2015**, 25, 7530-7538.

[10] D. D. Li, Y. Li, Z. Y. Xu, D. W. Wang, T. P. Wang, J. Zhao, H. H. Zhang, *Journal of Materials Science* **2018**, 53, 3647-3660.

[11] J. Zou, D. Xie, J. Xu, X. Song, X. Zeng, H. Wang, F. Zhao, *Applied Surface Science* **2022**, 571, 151322.

[12] K. Ma, F. Liu, M. Zhang, X. Zhang, J. P. Cheng, *Electrochimica Acta* **2017**, 225, 425-434.

[13] M. He, Y. He, X. Zhou, Q. Hu, S. Ding, Q. Zheng, D. Lin, X. Wei, *Dalton Transactions* **2021**, 50, 4643-4650.

[14] Y. Ren, H. Du, X. Zhou, Y. Liu, Q. Wang, S. Li, W. Wang, X. Dong, *Materials Today Energy* **2020**, 18, 100514.

[15] D. Zhang, X. Guo, X. Tong, Y. Chen, M. Duan, J. Shi, C. Jiang, L. Hu, Q. Kong, J. Zhang, *Journal of Alloys and Compounds* **2020**, 837, 155529.

[16] Z. Wang, W. Liu, J. Hu, W. He, H. Yang, C. Ling, Y. Xi, X. Wang, A. Liu, C. Hu, *Nano Energy* **2020**, 69, 104452.
